# The Evolutionary Portrait of Metazoan NAD Salvage

**DOI:** 10.1371/journal.pone.0064674

**Published:** 2013-05-28

**Authors:** João Carneiro, Sara Duarte-Pereira, Luísa Azevedo, L. Filipe C. Castro, Paulo Aguiar, Irina S. Moreira, António Amorim, Raquel M. Silva

**Affiliations:** 1 IPATIMUP-Institute of Molecular Pathology and Immunology of the University of Porto, Porto, Portugal; 2 Faculty of Sciences, University of Porto, Porto, Portugal; 3 Interdisciplinary Centre for Marine and Environmental Research (CIIMAR), CIMAR Associate Laboratory, University of Porto, Porto, Portugal; 4 CMUP - Centro de Matemática da Universidade do Porto, Porto, Portugal; 5 REQUIMTE - Rede de Química e Tecnologia, Faculty of Sciences, University of Porto, Porto, Portugal; University of Westminster, United Kingdom

## Abstract

Nicotinamide Adenine Dinucleotide (NAD) levels are essential for cellular homeostasis and survival. Main sources of intracellular NAD are the salvage pathways from nicotinamide, where Nicotinamide phosphoribosyltransferases (NAMPTs) and Nicotinamidases (PNCs) have a key role. NAMPTs and PNCs are important in aging, infection and disease conditions such as diabetes and cancer. These enzymes have been considered redundant since either one or the other exists in each individual genome. The co-occurrence of *NAMPT* and *PNC* was only recently detected in invertebrates though no structural or functional characterization exists for them. Here, using expression and evolutionary analysis combined with homology modeling and protein-ligand docking, we show that both genes are expressed simultaneously in key species of major invertebrate branches and emphasize sequence and structural conservation patterns in metazoan NAMPT and PNC homologues. The results anticipate that NAMPTs and PNCs are simultaneously active, raising the possibility that NAD salvage pathways are not redundant as both are maintained to fulfill the requirement for NAD production in some species.

## Introduction

Nicotinamide Adenine Dinucleotide (NAD) is an essential molecule to cells. As a cofactor in redox reactions, NAD regulates the metabolism and energy production and, as a substrate for NAD-consuming enzymes such as poly(ADP-ribose) polymerases (PARPs) and sirtuins, NAD is involved in DNA repair, transcriptional silencing and cell survival [1]. To maintain adequate NAD levels, several routes are used for NAD synthesis that depend on distinct precursors: *de novo* pathways synthesize NAD from tryptophan or aspartic acid whereas salvage pathways recycle NAD from nicotinamide (Nam), nicotinic acid (Na) and their ribosides [2]–[4].

The nicotinamide salvage pathway is the major source of intracellular NAD in humans [5], [6] and is also required for growth in several microorganisms [7]–[10]. NAD salvage from Nam is a two- or four-step reaction, in which the rate-limiting enzymes and functional homologues are, respectively, nicotinamide phosphoribosyltransferases (NAMPTs) and nicotinamidases (PNCs) [11]–[13]. In humans, *NAMPT* is widely studied due to its involvement in inflammation and disease such as cancer [14], [15]. In contrast, humans lack nicotinamidase but expression of the *Drosophila* Pnc protects human neuronal cells from death originated by oxidative stress [16]. Moreover, an increased Pnc1 and sirtuin activity confers protection to proteotoxic stress in yeast and *C. elegans* [17], [18]. The yeast Pnc1 is a biomarker of stress and a regulator of sirtuin activity [11], [18], and thus, most studies in yeast and invertebrates have focused in the link between these enzymes and aging [16], [19]. Notwithstanding, despite their importance to major cellular processes, there is a poor functional characterization of nicotinamidases [20], [21] and their role in infection has been less explored [7], [8], [22].

NAMPTs and PNCs act as regulators of enzymes from similar pathways, coordinating the overall metabolism and stress responses [23]. Moreover, both are pharmacologically relevant. NAMPT inhibitors are being used in clinical trials as anti-cancer agents [24]–[27] and nicotinamidases are attractive targets to the development of drugs for infectious diseases and anti-parasitic therapies [7], [8], [22], [28]–[30].

NAMPTs and PNCs do not co-occur in all organisms and, until very recently, lineages with both *NAMPT* and *PNC* had been only found in bacteria and algae [30]–[32]. *NAMPT* was thought to be absent from invertebrates but the discovery that *NAMPT* homologues are present in several invertebrate species and that some species have both *NAMPT* and *PNC* homologues [33] challenged the classical view that these enzymes are redundant and mutually exclusive [1], emphasizing the need for studies characterizing the structural and functional properties of these enzymes.

Motivated by the lack of information for *NAMPT* and *PNC* homologues in relevant invertebrate species, which would render the biological meaning of simultaneous *versus* unique occurrence of these proteins more evident, we carried out an integrated study to establish gene expression, amino acid conservation and structural comparisons. We provide experimental evidence that both genes are expressed simultaneously in key invertebrate species. In addition, evolutionary conserved patterns at the amino acid sequence and at the structural levels were detected. Also, using homology modeling and protein-ligand docking, we identify the amino acids that bind Nam in the active sites of invertebrate NAMPTs and PNCs. Taken together, the results suggest that invertebrate NAMPTs and PNCs are concurrently functional and, thus, that both NAD salvage pathways might not be redundant.

## Results

### Expression of invertebrate NAMPTs and PNCs

**Figure 1:**
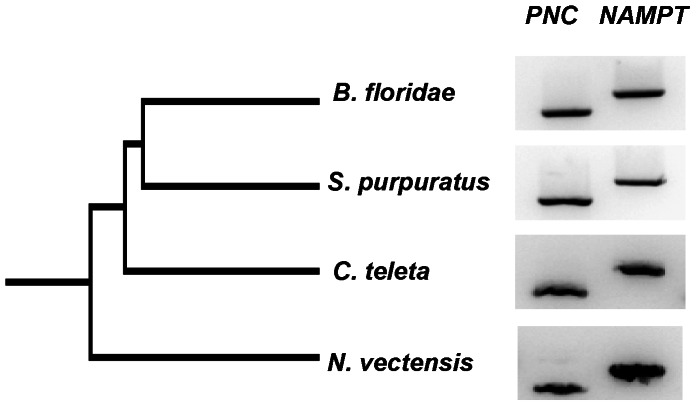
NAMPT and PNC homologues are co-expressed in invertebrates. RT-PCR analysis shows that NAMPT and PNC are simultaneously expressed in *Branchiostoma floridae*, *Strongylocentrotus purpuratus*, *Capitella teleta* and *Nematostella vectensis*.

*NAMPT* homologues have been previously found in the vibriophage KVP40 [34], bacteria [10], [32], and the unicellular green algae *Chlamydomonas reinhardtii* [31], motivating the search for *NAMPT* homologues in invertebrates, some of which simultaneously have *PNC* sequences [33] (Table S1). No recognizable *NAMPT* homologue has been detected so far in representative species of the phyla Arthropoda or Nematoda, although *NAMPT* and *PNC* were found in more basal lineages such as the choanoflagellate *Monosiga brevicollis* and the sea anemone *N. vectensis* (Figure 1). Such phylogenetic distribution is consistent with a scenario where both genes were present in the Metazoan ancestor and were selectively lost in specific lineages, as evidenced by the different patterns in protostomes. Namely, both genes were found in lophotrochozoans that includes mollusks (*Lottia gigantea*) and annelids (*C. teleta* and *Helobdella robusta*), and the absence of *NAMPT* was observed in ecdysozoans such as nematodes and arthropods. In deuterostomes, which comprises chordates, hemichordates and echinoderms, both genes were likely present in early lineages, which is supported by the evidence from the extant *B. floridae*, *Saccoglossus kowaleskii* and *S. purpuratus* species, but *NAMPT* was secondarily lost in the urochordate *Ciona intestinalis* while *PNC* was lost in vertebrates (Figure S1). RT-PCR of selected species showed that both *NAMPT* and *PNC* genes are expressed in the adult forms of *Branchiostoma floridae* (Cephalochordata), *Strongylocentrotus purpuratus* (Echinodermata), *Capitella teleta* (Annelida) and *Nematostella vectensis* (Cnidaria) (Figure 1). In addition, available EST (Expressed Sequence Tag) data indicates that *NAMPT* and *PNC* genes are also co-expressed during developmental stages (Table S2), suggesting a widespread usage of both Nam salvage pathways across Metazoans.

### Evolutionary divergence of NAMPTs and PNCs

**Figure 2:**
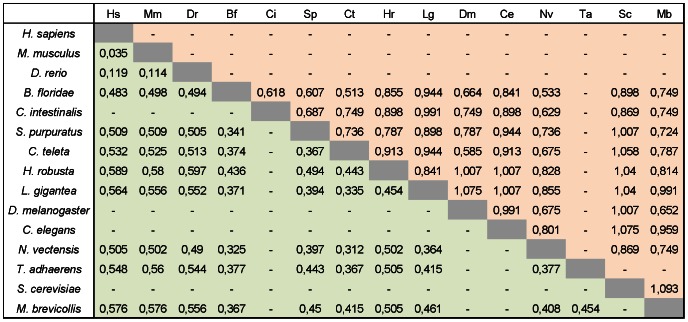
Evolutionary divergence between NAMPT and PNC homologues. The estimates of evolutionary divergence were calculated as amino acid substitutions per site between NAMPT (green) and PNC (orange) sequences for several species. Hs, *Homo sapiens*; Mm, *Mus musculus*; Dr, *Danio rerio*; Bf, *Branchiostoma floridae*; Ci, *Ciona intestinalis*; Sp, *Strongylocentrotus purpuratus*; Ct, *Capitella teleta*; Hr, *Helobdella robusta*; Lg, *Lottia gigantea*; Dm, *Drosophila melanogaster*; Ce, *Caenorhabditis elegans*; Nv, *Nematostella vectensis*; Ta, *Trichoplax adhaerens*; Sc, *Saccharomyces cerevisiae*; Mb, *Monosiga brevicollis*.

We have further characterized the evolutionary divergence of NAMPT and PNC homologues, measured as protein distances calculated from amino acid sequence alignments (Figure 2). The resulting matrix (Figure 2) showed that NAMPT is conserved, even when large evolutionary distances are considered. For example, the divergence between the human and cnidarian (*N. vectensis*) NAMPT homologues is about 50%, as much as when compared with amphioxus (*B. floridae*). Among invertebrates the sequences showing the smallest divergence are from *N. vectensis* and *C. teleta* (31.2%). Conversely, PNC sequences are highly divergent even in closely related species, as shown for the annelids *C. teleta* and *H. robusta*, or the basal chordates *B. floridae* and *C. intestinalis*. Curiously, the smallest divergence between PNC sequences was found for *C. teleta* and *B. floridae* (51.3%). This trend was also evident when we plotted protein distances taking implicitly in consideration the evolutionary divergence time between each pair of species studied (Movie S1 and Table S3). Analyses of protein distances (*pd*) indicated that NAMPT homologues are considerably more conserved (*pd* = 0.447±0.116) than PNC (*pd* = 0.842±0.151) (mean±std), which is remarkable for species spanning over 1000 million years of divergence (Table S3). For PNC proteins, in addition to the larger values, no correlation with evolutionary distance was observed, while NAMPT distances were smaller and increased consistently with the evolutionary distance (*ed*) between species. The Kendall rank correlation coefficient was used to measure the dependence between *pd* and *ed*, showing no relevant dependence between both quantities for PNC (τ = −0.052). However, for NAMPT both quantities vary consistently (τ = 0.413).

### Motif conservation in NAMPTs and PNCs

**Figure 3:**
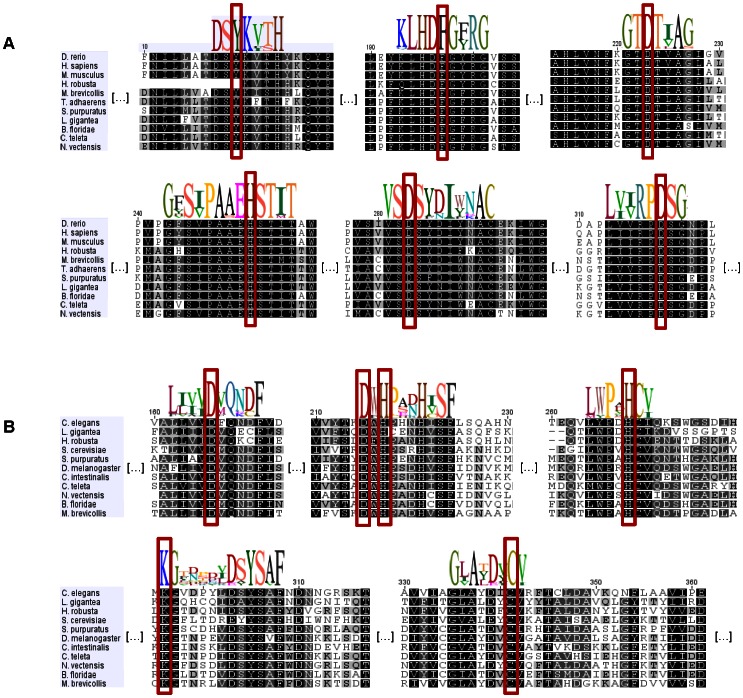
Amino acid motifs found in NAMPT and PNC homologues. The conserved amino acid motifs surrounding the active site residues (boxed) are shown as logos and displayed above the aligned sequences. NAMPT conservation is highlighted by the large blocks of identical amino acids that are found in the species analyzed (**A**). In PNC homologues, although the overall amino acid identity is low, the presence of conserved motifs is still detected throughout the species analyzed that range wide evolutionary distances (**B**).

We next used the previously constructed amino acid sequence alignments dataset to search for conserved motifs in NAMPT and PNC homologues. In line with the aforementioned results, analyses of NAMPT sequences (Figure 3A) revealed conserved amino acid motifs surrounding catalytic residues [24], [25], [35]–[37] Tyr18, Phe193, Asp219, His247, Asp279, Asp313, corresponding to the boxed amino acids in Figure 3. As well, Asp16 and Arg311, Gly353 and Asp354, and Gly384 that bind nicotinamide, ribose or phosphate, respectively, are preserved and the additional NMN interacting residues Arg196 and Gly383 in rat NAMPT [25] are present in all sequences analyzed. The amino acid stretches that represent the dimer interface are also conserved in invertebrate NAMPTs (Figure 3A and Figure S2), as previously shown for vertebrates [25].

Similar analyses on PNC homologues showed that, while overall amino acid sequence identity is low (Figure 3B), motifs surrounding metal-binding and catalytic residues (boxed amino acids) show up. Indeed, all PNC sequences have conserved residues that coordinate the metal ion (corresponding to *Saccharomyces cerevisiae* Asp51, His53 and His94) and the catalytic triad (*S. cerevisiae* Asp8, Lys122 and Cys167). The characteristic *cis*-peptide bond that has been identified in available nicotinamidase/pyrazinamidase structures also corresponds to conserved residues present in these species, namely Val-Ala in *Pyrococcus horikoshii, S. cerevisiae, Leishmania infantum* and *C. intestinalis* [7], [38], [39], Ile-Ala in *Mycobacterium tuberculosis, Acinetobacter baumanii, H. robusta* and *B. floridae* [40], [41], or Val-Leu in *Streptococcus pneumoniae* [42], and are preceded by a conserved glycine that has a role in catalysis [38], [40], [41]. Additionally, mutations that lead to *M. tuberculosis* loss of pyrazinamidase activity have defined residues that delineate the active site scaffold [38], corresponding to *S. cerevisiae* Glu10, Asp12, Phe13, Leu20, His57, Trp91, Gly123, Tyr131, Ser132, Val162, Ala163, Tyr166 and Thr171, and most of them are conserved in all invertebrate PNC sequences as well (Figure 3B and Figure S3).

### Genetic architecture conservation of *NAMPT* homologues

**Figure 4:**
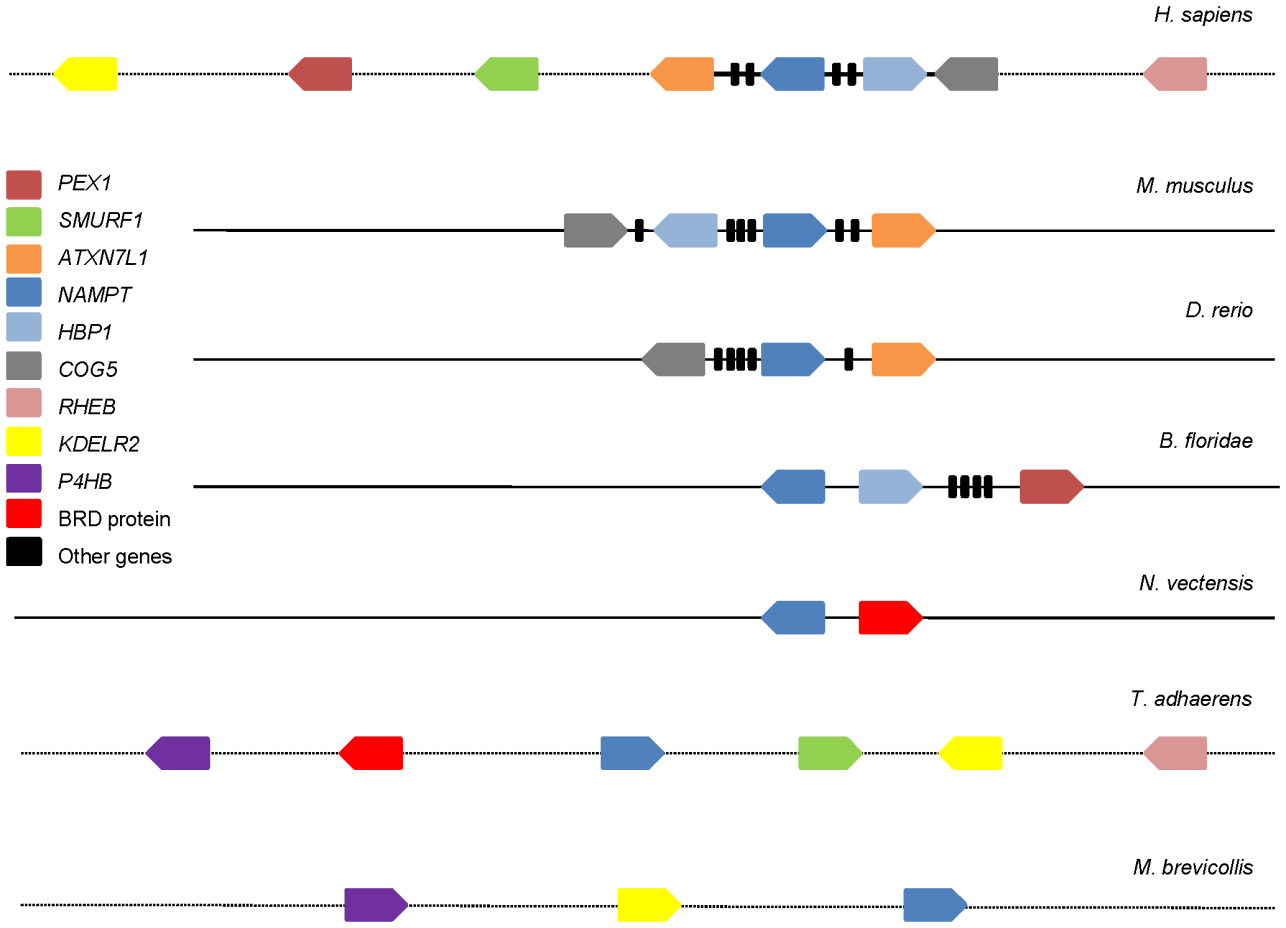
Syntenic organization of NAMPT homologues. Gene order and organization are represented for several lineages, and show conservation of microsynteny in chordates. *H. sapiens* chromosome 7, *M. musculus* chromosome 12, *D. rerio* chromosome 4, *B. floridae* scaffold 633, *N. vectensis* scaffold 360, *T. adhaerens* scaffold 2 and *M. brevicollis* scaffold 7 are displayed and dots indicate intervals containing multiple genes (>4).

Given the degree of conservation of both proteins, and taking into account the divergence times of over 1000 million years between the species considered here, we have investigated the conservation at the gene structure and genome organization levels. *NAMPT* retained microsynteny in chordates, as indicated by the conserved gene order between *H. sapiens*, *M. musculus*, *D. rerio* and *B. floridae*, and also showed macrosynteny conservation in some lineages, namely between *Trichoplax adhaerens* and either *H. sapiens*, *N. vectensis* or *M. brevicollis* (Figure 4 and Figures S4, S5). For *PNC* homologues, no syntenic regions were found. Although recent studies point to a higher level of microsynteny conservation in metazoans than previously estimated [43], these evidences are challenging in some lineages due to poor genome annotation and breakdown in small scaffolds. At the level of exon-intron structure, *NAMPT* is more homogeneous than *PNC*, considering the number and size of exons, and total gene length (Figures S6, S7). The exception is observed in *N. vectensis*, where *NAMPT* has many small exons spanning 14 Kb in the genome, while *PNC* has only two exons in less than 2 Kb. Using the information on conserved motifs and gene structure, we were able to successfully identify *NAMPT* and *PNC* homologues as well as predict the corresponding gene structures in the hemichordate *S. kowaleskii*, a phylogenetic informative species (Figure S8).

### Secondary structure conservation of *PNC* homologues

**Figure 5:**
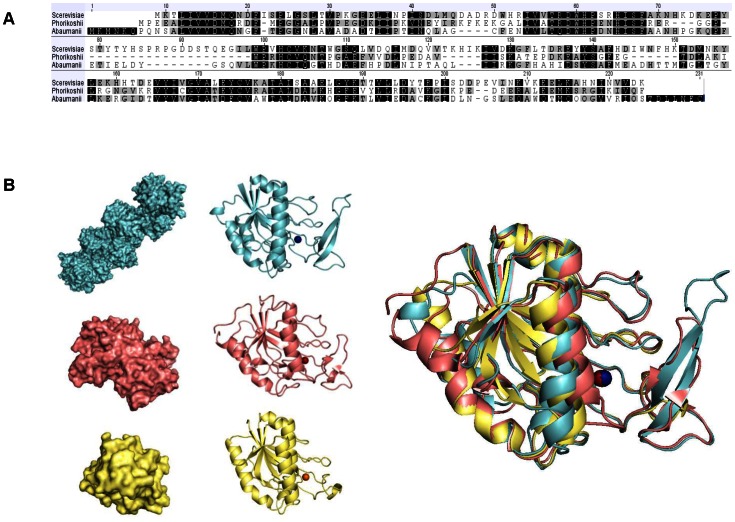
Structural conservation between PNC homologues. Alignment of sequences (**A**) and structures (**B**) of PNC homologues from *P.horikoshii* (yellow), *A.baumanii* (pink) and *S.cerevisiae* (blue). Although there is an increasing structural complexity from a monomer in Archaea, a dimer in Bacteria and an heptamer in Fungi and the amino acid identity of the sequences is around 30%, the 3D structural subunits of PNC homologues are superimposable.

**Figure 6:**
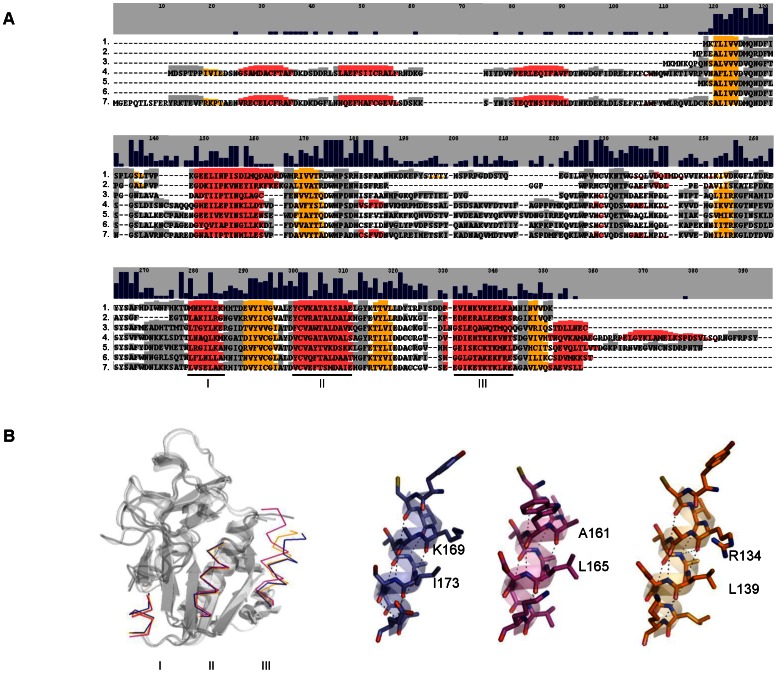
Predicted secondary structure of PNC homologues. (**A**) Aligned amino acid sequences of representative PNC homologues are displayed in function of the secondary structure. Alpha-helices are shown in red, beta-sheets are in yellow and grey represents coiled regions. Regions of structural conservation are highlighted in color even when the primary sequences are not conserved as demonstrated by the graphic bars above the sequences. 1, *Saccharomyces cerevisiae*; 2, *Pyrococcus horikoshii*; 3, *Acinetobacter baumanii*; 4, *Drosophila melanogaster*; 5, *Ciona intestinalis*; 6, *Nematostella vectensis*; 7, *Branchiostoma floridae*. (**B**) Alpha-helices I, II and III formed by groups of unrelated amino acids are structurally equivalent as shown by the 3D superimposition. In blue, *S. cerevisiae*; in pink, *A.baumanii*; and in yellow, *P. horikoshii*.

Nicotinamidase sequences are poorly conserved even in closely related species (Figures 2 and 3). Yet, considering some structures determined for archaea (*P. horikoshii*, PDB id: 1IM5), bacteria (*A. baumanii*, PDB id: 2WTA) and fungi (*S. cerevisiae*, PDB id: 2H0R), sharing only 30% protein identity (Figure 5A), the 3D structures are perfectly superimposable (Figure 5B). Such structural conservation is observed across the three domains of life, as all PNC enzymes share a similar core fold (Figure S9), with a potential increase in complexity of the enzyme that is active as a monomer in *P. horikoshii* [38], dimer in *A. baumanii* [40] and heptamer in *S. cerevisiae* [39]. Thus, we have aligned PNC sequences based on secondary structure predictions and determined that invertebrate PNCs also show structural conservation (Figure 6). The regions corresponding to alpha-helices (red) and beta-sheets (yellow) are conserved at the structural level, even if the amino acids are not (Figure 6A). For instance, the alpha-helices of regions I, II and III comprise different amino acids while displaying a similar fold. To illustrate this, region II is shown in detail for *P. horikoshii*, *A. baumanii* and *S. cerevisiae* (Figure 6B).

### Modeling and docking analyses of invertebrate NAMPTs and PNCs

**Figure 7:**
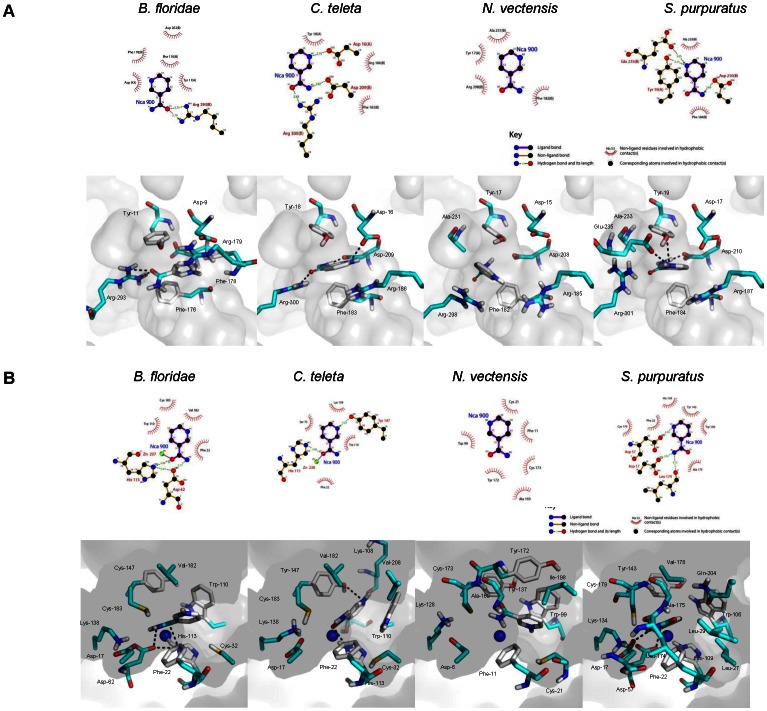
Hydrophobic interactions and hydrogen bonding network between the ligand (NCA) and the various receptors. NAMPT (A) and PNC (B) representations in LIGPLOT (upper panels) and PyMOL (lower panels) representations are shown for *Branchiostoma floridae*, *Capitella teleta*, *Nematostella vectensis*, and *Strongylocentrotus purpuratus*. The major binding determinants are represented in cyan stick. The Zn^2+^ atom is in blue vdW representation.

To gain insight into the structures of invertebrate NAMPTs and PNCs, we have performed homology modeling and protein-ligand docking. To overcome limitations in the interpretation of results, we have used several templates to generate the models (Table S4). The LIGPLOT program was used to generate schematic diagrams between ligand (Nicotinamide, NCA) and receptor (NAMPT and PNC), which are shown in Figure 7. The prediction accuracy redocking test performed for the NAMPT (PDB 2E5D from *H. sapiens*) and PNC (PDB 3R2J from *L. infantum*), were in agreement with the ligand-receptor conformation in these X-ray structures. We obtained a similar active site ligand-receptor interaction for both NAMPT and PNC, which insure that the docking approach was accurate enough to be applied to the various molecular systems.

In NAMPT protein active site, all species, except *N. vectensis*, maintained most of the ligand-receptor interactions when compared with the structure of human NAMPT (Figure 7A). The homologous NAMPT of *B. floridae* has a hydrogen bond network that stabilizes the active site with two H-bonds between the side-chain of Arg-293 and the oxygen atom of the ligand. A similar bonding network can be observed in the human protein (PDB 2E5D) where Asp-219 binds to the nitrogen atom of the substrate (NCA). Hydrophobic interactions are similar when compared with the human active site. In *C. teleta*, H-bond interactions between Arg-300 and NCA oxygen moiety and between Asp-209 and Asp-16 to both NCA nitrogens preserve the NCA conformation in the active site. Two hydrophobic interactions in *C. teleta* (Tyr-18 and Phe-183) with ligand atoms are not seen. In *N. vectensis* no H-bond interaction is present, but the most important hydrophobic interactions, Phe-182(B), Arg-298(B) and Tyr-17(A), are preserved. The H-bond interaction network of *S. purpuratus* shows that Asp-210(B) H-bond is maintained. Two other H-bonds, Tyr-19(A) and Glu-235(B), and hydrophobic interactions of the NCA ligand to Phe-184 (B) and Ala-233 (B) are also present. Globally, the NAMPT binding modes obtained by docking for the species analyzed shared the critical hydrophobic and hydrogen bonding interactions and, if not (e.g. *N. vectensis*), the conformational status of NCA was maintained.

Next we also analyzed the conformational changes of PNC active and catalytic sites (flexible residues) in the four species (Figure 7B). In the *B. floridae* PNC, Phe-22, Trp-110, Val-182 and Cys-183 hydrophobic interactions contribute to the binding status of NCA. The three hydrogen bonding interactions (His-113 to NCA oxygen atom, Asp-62 to NCA nitrogen atom and His-113 to Asp-62) sustain the conformational position of the ligand. The Zn^2+^ keeps the strong binding to the ligand that was also present in *L. infantum* PNC (PDB 3R2J). In *C. teleta* the Trp-110 (hydrophobic interaction) and Tyr-147 (H-bond interaction) are the residues from the active site that play an important role in the ligand-receptor interaction. As in *B. floridae*, the His-113 has a hydrogen bond connection with NCA. Two other H-bond interactions not present in *L. infantum* PNC (Ser-70 and Lys-108) appear to be important to ligand binding. The interaction between Zn^2+^ and ligand is maintained. Although no significant changes in ligand conformation were observed, hydrogen bonds in *N. vectensis* were not predicted. When compared to 3R2J, hydrophobic interactions Cys-21, Trp-99, Ala-169 and Cys-173 are kept for the active site residues. Hydrophobic interactions for the catalytic residue Cys-173 are present, as well as a newly arisen Phe-11 interaction with the ligand. In *S. purpuratus*, hydrophobic contacts between Tyr-106, Trp-143, Ala-175 and the ligand are retained. Catalytic site residues Asp-17 and Cys-179 also bind to the ligand through an H-bond and a hydrophobic interaction. Two unique hydrogen bonds (Asp-57 and Leu-174) and hydrophobic contacts (His-109 and Phe-22) arise in the ligand-protein interaction. It can also be noticed that a conserved histidine (His-113 in *B. floridae* and *C. teleta*, and His-109 in *S. purpuratus*) maintains the interaction with the ligand.

## Discussion

Nicotinamide phosphoribosyltransferases (NAMPTs) and nicotinamidases (PNCs) are the main NAD salvage enzymes and, until recently, were thought to occur in distinct lineages. Our data show that several Metazoan species have predicted homologues of both enzymes and that both genes are simultaneously expressed in *B. floridae, S. purpuratus*, *C. teleta* and *N. vectensis*. The distribution of NAMPT and PNC homologues points to the presence of both genes in early eukaryote evolution with selective gene loss and retention in different animal lineages. Interestingly, loss of either one of the genes was predominantly found in fast evolving lineages, namely *D. melanogaster*, *C. elegans* and *C. intestinalis*, while slow-evolving species such as *B. floridae* retained both [44]. This is also reflected in genome architecture, with conserved *NAMPT* microsynteny in vertebrates and *B. floridae*.

We also highlight different conservation patterns in NAMPT and PNC homologues, at the protein amino acid sequence and at the 3D structural level. NAMPT sequences are highly conserved, as evidenced by small evolutionary divergences between species and long stretches of identical amino acids surrounding important catalytic and structural positions. As dimerization is required for NAMPT activity [25], in addition to active site residues that interact with the substrates and reaction products, amino acids that constitute the dimer interface are also conserved. For PNCs the sequence identity is lower, yet, critical amino acids are conserved and the overall fold is maintained in all the three domains of life. These are unifying features of nicotinamidases, even though there is a diversity of catalytic mechanisms described, with some exceptions concerning metal binding and metal ion coordination [7], [20], [21], [29], [41], [42].

Homology modeling and protein-ligand docking indicates that active site residues and interactions of invertebrate NAMPTs with the substrate, nicotinamide, are similar to what is described for vertebrate NAMPTs [24], [25], [35]–[37]. In invertebrate PNCs, most interactions are maintained while additional hydrogen bonds and hydrophobic contacts were found. These new interactions might derive from complementary amino acid changes as a result of epistatic interactions between residues [21], [45], which is consistent with a structural conservation of PNCs.

Our analyses validate invertebrate NAMPTs and PNCs, suggesting that both the two-step and the four-step NAD salvage pathways are functional in these organisms. These findings imply that either these enzymes are not redundant, or that specific metabolic requirements call for increased NAD production in some species that only the presence of both enzymes would fulfill.

## Materials and Methods

### Sequence analysis

The human *NAMPT* and the yeast *PNC1* amino acid sequences were used as queries in BLAST searches [46], from National Center of Biotechnology Information, NCBI (http://www.ncbi.nlm.nih.gov/sites/genome) and Joint Genome Institute, JGI (http://genome.jgi-psf.org/) sequenced genomes. In organisms with multiple hits, the reciprocal best hit was selected for further analysis. All sequences retrieved in this process and further analyzed are listed in Table S1. Estimates of evolutionary divergence between sequences were conducted in MEGA5 [47] and calculated as the number of amino acid substitutions per site. Analyses were conducted using the Poisson correction model [48] and involved 13 amino acid sequence homologues for each protein. Positions containing gaps and missing data were eliminated, resulting in a total of 436 (NAMPT) and 167 (PNC) positions in the final dataset. Alignments were visualized in Geneious [49] v5.5.6 to generate logos. Structural alignments of PNC homologues were performed in Ali2D (http://toolkit.tuebingen.mpg.de/ali2d). Divergence times between species were estimated using Time Tree (http://www.timetree.org/) [50]. MATLAB version R2010b was used to generate 3D graphics (the input data is shown as Table S3) and calculate Kendall rank correlation coefficients. Correlations were measured against a reference function consisting of a monotonic increasing function of protein distances against evolutionary divergence (the hypothesis). Synteny was determined using the CHSminer software (http://www.biosino.org/papers/CHSMiner/) [51] and the JGI genome portal (http://genome.jgi-psf.org/). *Saccoglossus kowaleskii* BLAST searches were also performed as described above (http://blast.hgsc.bcm.tmc.edu/blast.hgsc?organism=20), the corresponding genome contigs (115790 and 40985) were retrieved and the NAMPT and PNC protein sequences were manually predicted, based on the conserved motifs identified. Exon predictions were then performed in Genescan (http://genes.mit.edu/GENSCAN.html).

### Molecular homology modeling

Prime [52] was used to search homologous proteins in NCBI PDB database (http://www.rcsb.org/pdb/home/home.do) for PNC and NAMPT. PDB templates (Table S4) were selected considering lowest e-values (<1×10−6), and structures without many missing residues (gaps<20%). PNC and NAMPT sequences for the species *B. floridae*, *C. teleta*, *S. purpuratus* and *N. vectensis* were used to generate the alignments with homologue proteins. For secondary structure prediction the third-party program SSpro [53], [54] was used and then all the templates re-aligned to the query sequence. The resulting alignment was used to build the protein models. The LigPrep [55] interactive optimizer (protein preparation wizard) with neutral pH was used to optimize the protein model. Finally hydrogens were added, bond order was assigned and selenomethionines were converted to methionines for the generated models.

### Molecular docking simulations

The 3D-structures of ligands were obtained from the PDB structures. The protein-ligand complexes were prepared with AutoDockTools [56], [57]: hydrogen atoms were added for each protein and Kollman united atom charges assigned. Hydrogens were also added to the ligand (NCA) and charges were calculated by the Gasteiger-Marsili method. The rotatable bonds in the ligands were assigned with AutoDockTools. The Zn atom of PNC was assigned a charge of +2. AutoDock4.2 [56] was used to perform protein-ligand docking calculations. To insure the accuracy of our methodological approach we first have done redocking of the two most recently available X-ray structures (NAMPT PDB 2E5D and PNC PDB 3R2J) and then applied it to the various predicted protein-ligand systems. Various grid sizes were tested using as structural criteria the similarity between our docked results and the X-ray structure of *H. sapiens* NAMPT (2E5D) and *L. infantum* PNC (3R2J). We have selected a cubic grid box of 30×25×40 Å for NAMPT and 35×35×40 Å for PNC, centered on the C2–C5 ligand atoms distance mean with a grid spacing of 0.375 Å as shown in Table S4.

We considered the binding pockets described in the literature [7], [36] (also shown in Table S5) to perform the flexible protein-ligand docking. The corresponding residues in the homology alignment are described in Table S5. We performed the docking simulations using 100 independent Lamarckian genetic algorithm (LGA) runs, with the population size set to 200, the number of energy evaluations set to 10 000 000 and the maximum number of generations set to 27 000. All other parameters were used as default [58], [59]. The results were analysed clustering together the conformations within a RMSD of 2 Å. The cluster with lower energy and with a conformation similar to the X-ray structure of NAMPT (PDB id: 2E5D) and PNC (PDB id: 3R2J) was selected for each species.

### H-bonds and hydrophobic interactions for ligand-receptor molecules

Interactions between the ligand (NCA) and receptors (NAMPT and PNC) were calculated using LIGPLOT [60]. The hydrogen bonds were calculated using geometrical criteria [61] of protein-ligand complex (The used criteria is: H–A distance <2.7 Å, D–A distance <3.3 Å, D–H–A angle >90°, D–A–AA angle >90° and H–A–AA angle >90°, where A is the hydrogen acceptor, D is the hydrogen donor, AA is the atom attached to the hydrogen acceptor, and H an atom of hydrogen). LIGPLOT also calculates non-covalent bond interactions (hydrophobic interactions) by applying a simple cut-off of 3.9 Å. LIGPLOT diagrams were generated for each species. PyMOL [62] was used to generate the 3D images.

### Expression analysis

*B. floridae* (whole organism), *C. teleta* (whole organism), *S. purpuratus* (gonad) and *N. vectensis* (whole organism) samples were obtained from Ocean Genome Legacy (OGL Accession ID numbers S13045, S13061, S13034 and S13115, respectively) [63]. RNA was extracted with the Illustra TriplePrep kit (GE Healthcare) and genomic DNA was removed from RNA preparations with an additional DNase treatment using DNase I, RNase-free (Fermentas, Thermo Fisher Scientific Inc.), according to the manufacturer's procedure. Complementary DNA (cDNA) was synthesized from 1 µg of total RNA using the RETROscrip® First Strand Synthesis Kit (Ambion) with oligo-dT primers according to the manufacturer's instructions. Reverse-transcription PCR reactions were prepared using HotStarTaq® Master Mix Kit (Qiagen) with 2 µl of the synthesized cDNA in a 10 µl final volume. Q solution was included in the reaction (10%) in NAMPT amplification in *B. floridae* and *S. purpuratus*. PNC and NAMPT were amplified with species-specific primers described in Table S6, with a final concentration of 0.2 µM. Thermocycling conditions were as follows: initial denaturation at 95°C for 15 min, 40 cycles at 95°C for 30 sec, variable annealing temperatures ranging from 52°C to 62°C (Table S6) for 1 min30 sec, and 72°C for 1 min, and a final extension step of 10 min at 72°C. All amplification products were visualized on 1.5% agarose gels and were confirmed by sequencing. For that, PCR products were purified with ExoSAP-IT (USB Corporation) by incubation at 37°C for 15 min, followed by enzyme inactivation for 15 min at 85°C. The resulting purified fragments were sequenced using an ABI Big Dye Terminator Cycle Sequencing Ready Reaction kit v 3.1 (Applied Biosystems) and analyzed in an ABI PRISM 3130xl (Applied Biosystems).

Expressed Sequence Tag (EST) information was retrieved from available databases for *B. floridae* [64], *S. purpuratus* [65] and *N. vectensis* [66] and is detailed in Table S2.

## Supporting Information

Movie S1 **Evolutionary divergence between NAMPT and PNC homologues.** Protein distances were plotted for each pair of species arranged accordingly to their respective divergence time. This plot shows that NAMPT is highly conserved across large evolutionary distances, while PNC is less conserved even in closely related species. Notice that in addition to being highly conserved, protein distances and evolutionary distances are correlated in NAMPT (quantified by the Kendall coefficient of 0.413), as opposed to PNC (where the Kendall coefficient was −0.052).
(MP4)

Figure S1 **Distribution of** ***NAMPT*** **and** ***PNC*** **homologues across the tree of life.** The presence or absence of *NAMPT* and *PNC* sequences is indicated on the columns by a plus or a minus. Protostomes are divided in ecdysozoans and lophotrochozoans (green and blue boxes of the tree, respectively), while Deuterostomes are represented in the red box.
(TIF)

Figure S2 **Alignment of the amino acid sequences from NAMPT homologues.** Catalytic residues are marked with red dots and residues that bind nicotinamide, ribose, phosphate or NMN are highlighted in blue.
(TIF)

Figure S3 **Alignment of the amino acid sequences from** ***PNC*** **homologues.** Catalytic residues are marked with red dots and residues that bind zinc are highlighted in blue. Additional residues of the active site are shown in green.
(TIF)

Figure S4 **Vertebrate** ***NAMPT*** **synteny.** Conserved synteny blocks detected between the Human, Mouse and Zebrafish genomes. Input data was automatically retrieved from Ensembl release 64 using CHSminer. Corresponding chromosomes are indicated.
(TIF)

Figure S5 **Invertebrate** ***NAMPT*** **synteny.** Synteny blocks were retrieved from JGI and represent *T. adhaerens* versus *H. sapiens* (A), *M. brevicollis* (B), and *N. vectensis* (C) genomes. Corresponding scaffolds and chromosomes are indicated.
(TIF)

Figure S6 ***NAMPT*** **gene structure.** Exon-intron predictions were performed in Gene Structure Display Server (http://gsds.cbi.pku.edu.cn/).
(TIF)

Figure S7 ***PNC*** **gene structure.** Exon-intron predictions were performed in Gene Structure Display Server (http://gsds.cbi.pku.edu.cn/).
(TIF)

Figure S8 ***NAMPT*** **(A) and** ***PNC*** **(B) amino acid alignments indicating exon size and number.** *Saccoglossus kowaleskii* (Sk) protein sequences were manually predicted, based on the conserved motifs identified in this paper. Exon predictions were performed in Genescan (http://genes.mit.edu/GENSCAN.html).
(TIF)

Figure S9 **PNC alignments of all the templates available.** (A) Alignments of the amino acid sequences. (B) Structural alignments of 2H0R (blue), 1IM5 (yellow), 2WTA (pink), 3R2J (grey), 3PL1 (purple) and 3O90 (green). (C) RMSD scores of the structural alignments. All structures are superimposed on the right.
(TIF)

Table S1 **Sequences of NAMPT and PNC orthologues.** The table lists the species and the associated protein identifier and source used in this work.
(XLSX)

Table S2 **EST sequences of NAMPT and PNC.** The table lists the species with available EST data and the associated identifier and source.
(XLSX)

Table S3 **MATLAB input data.** For each pair of species, mean divergence times are shown in million years as well as protein distances calculated from NAMPT and PNC amino acid alignments. −100 was the value assigned for the cases where one of the proteins is absent from the species pair.
(XLSX)

Table S4 **PDBs used as reference and grid parameters for the docking calculations.**
(XLSX)

Table S5 **Residue positions for each species that correspond to the reference residues (NAMPT PDB id 2E5D and PNC PDB id 3R2J) of the alignment used to model the proteins.**
(XLSX)

Table S6 **Oligonucleotide sequences used for amplification of NAMPT and PNC from** ***B. floridae*** **,** ***S. purpuratus*** **,** ***C. teleta*** **and** ***N. vectensis*** **.**
(XLSX)
