# Supplementary figures and images for: The Evolutionary Portrait of Metazoan NAD Salvage
Source: PLoS One. 2013 May 28;8(5):e64674. doi: 10.1371/journal.pone.0064674 (PMC3665594; doi:10.1371/journal.pone.0064674)

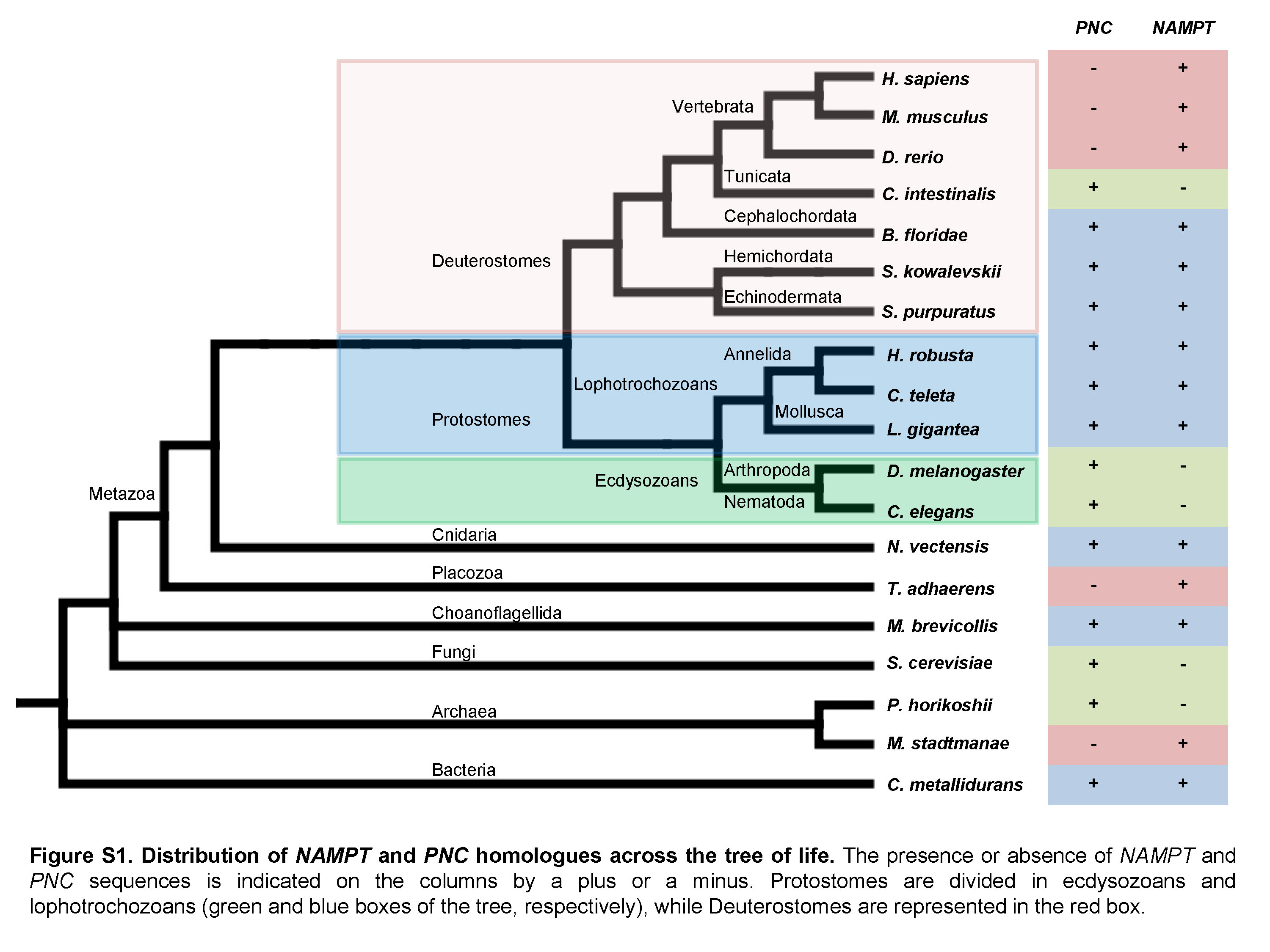

Supplement: Figure S1 — Distribution of NAMPT and PNC homologues across the tree of life. The presence or absence of NAMPT and PNC sequences is indicated on the columns by a plus or a minus. Protostomes are divided in ecdysozoans and lophotrochozoans (green and blue boxes of the tree, respectively), while Deuterostomes are represented in the red box. (TIF) [file pone.0064674.s002.tif]

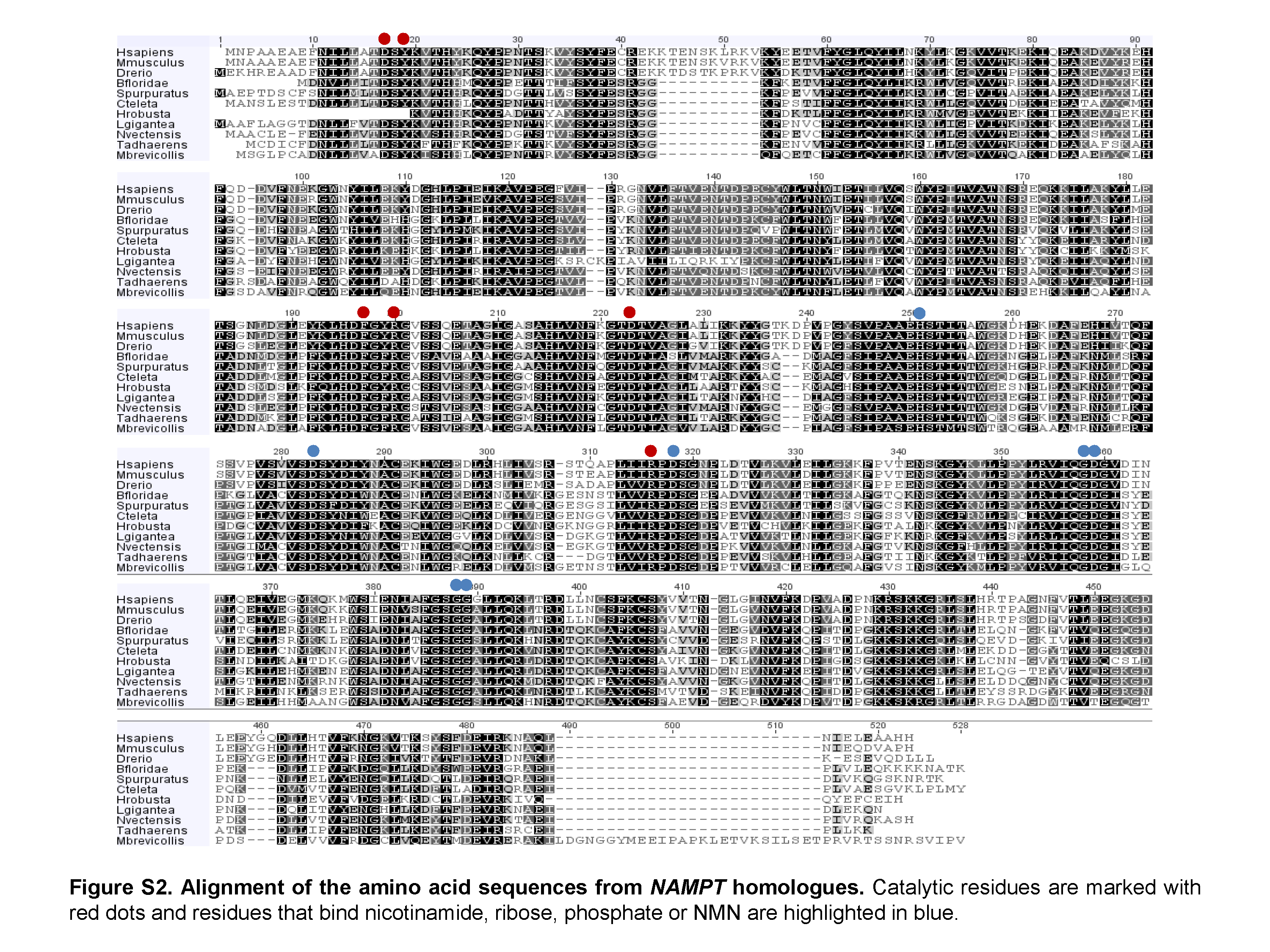

Supplement: Figure S2 — Alignment of the amino acid sequences from NAMPT homologues. Catalytic residues are marked with red dots and residues that bind nicotinamide, ribose, phosphate or NMN are highlighted in blue. (TIF) [file pone.0064674.s003.tif]

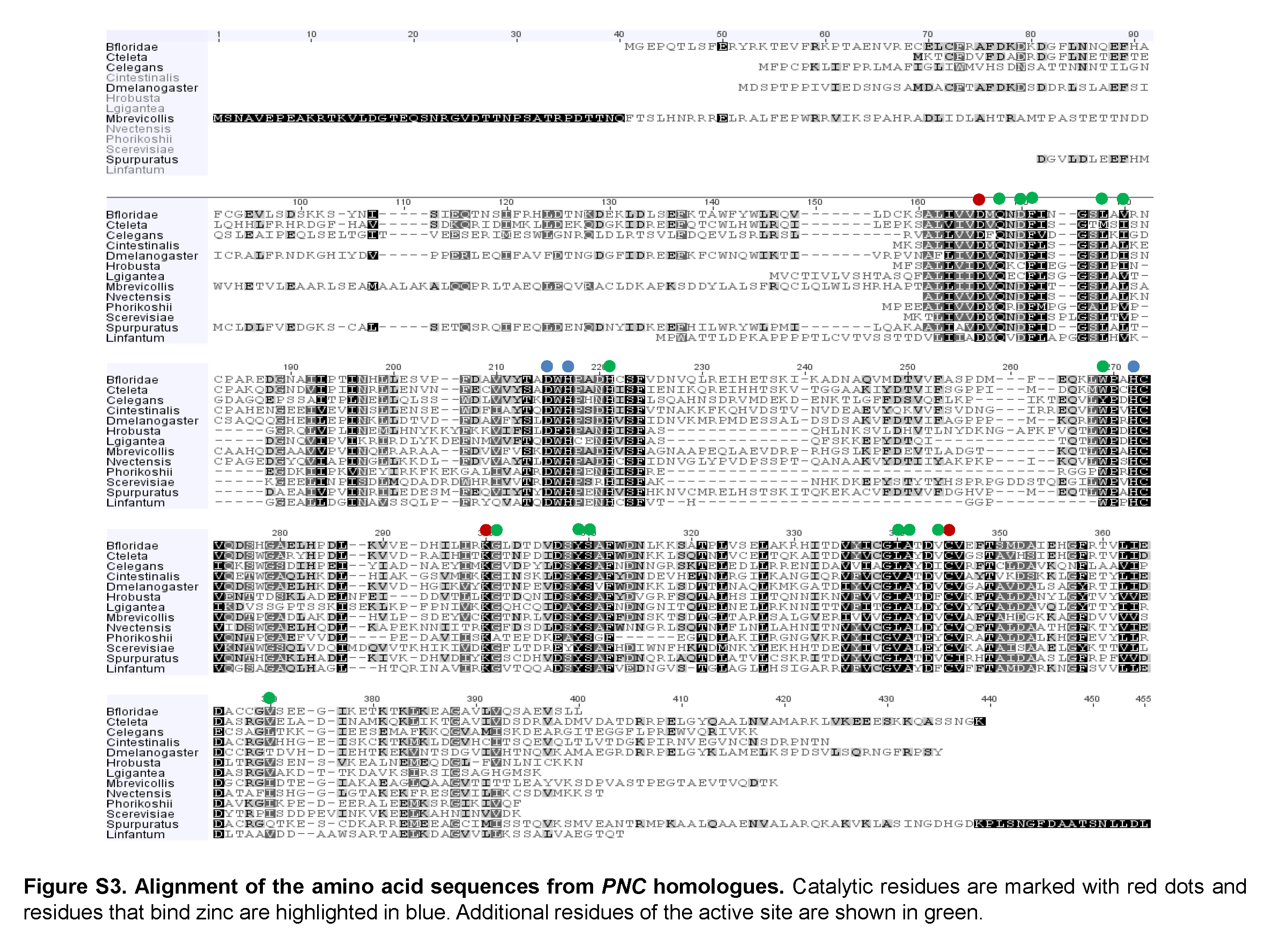

Supplement: Figure S3 — Alignment of the amino acid sequences from PNC homologues. Catalytic residues are marked with red dots and residues that bind zinc are highlighted in blue. Additional residues of the active site are shown in green. (TIF) [file pone.0064674.s004.tif]

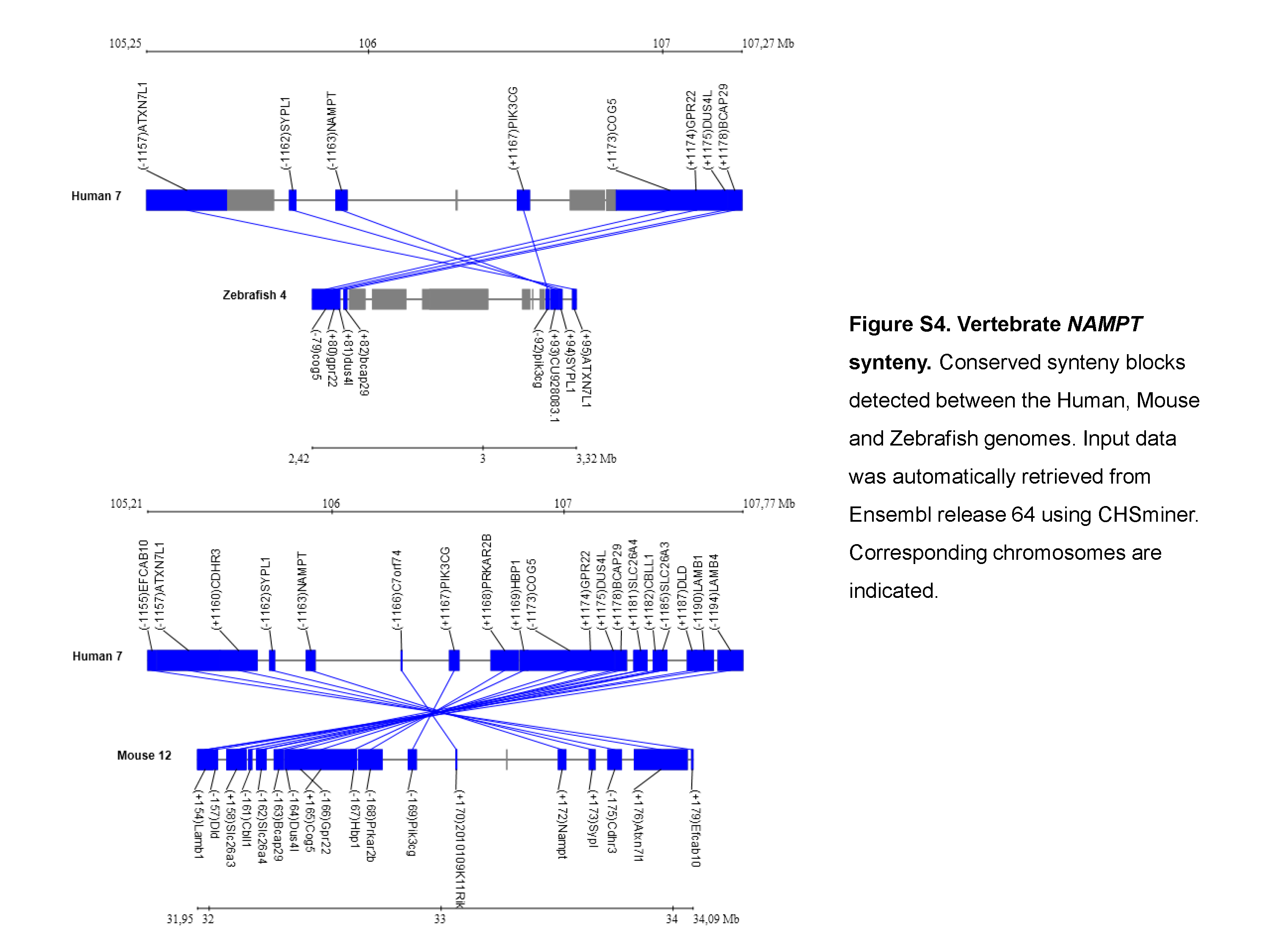

Supplement: Figure S4 — Vertebrate NAMPT synteny. Conserved synteny blocks detected between the Human, Mouse and Zebrafish genomes. Input data was automatically retrieved from Ensembl release 64 using CHSminer. Corresponding chromosomes are indicated. (TIF) [file pone.0064674.s005.tif]

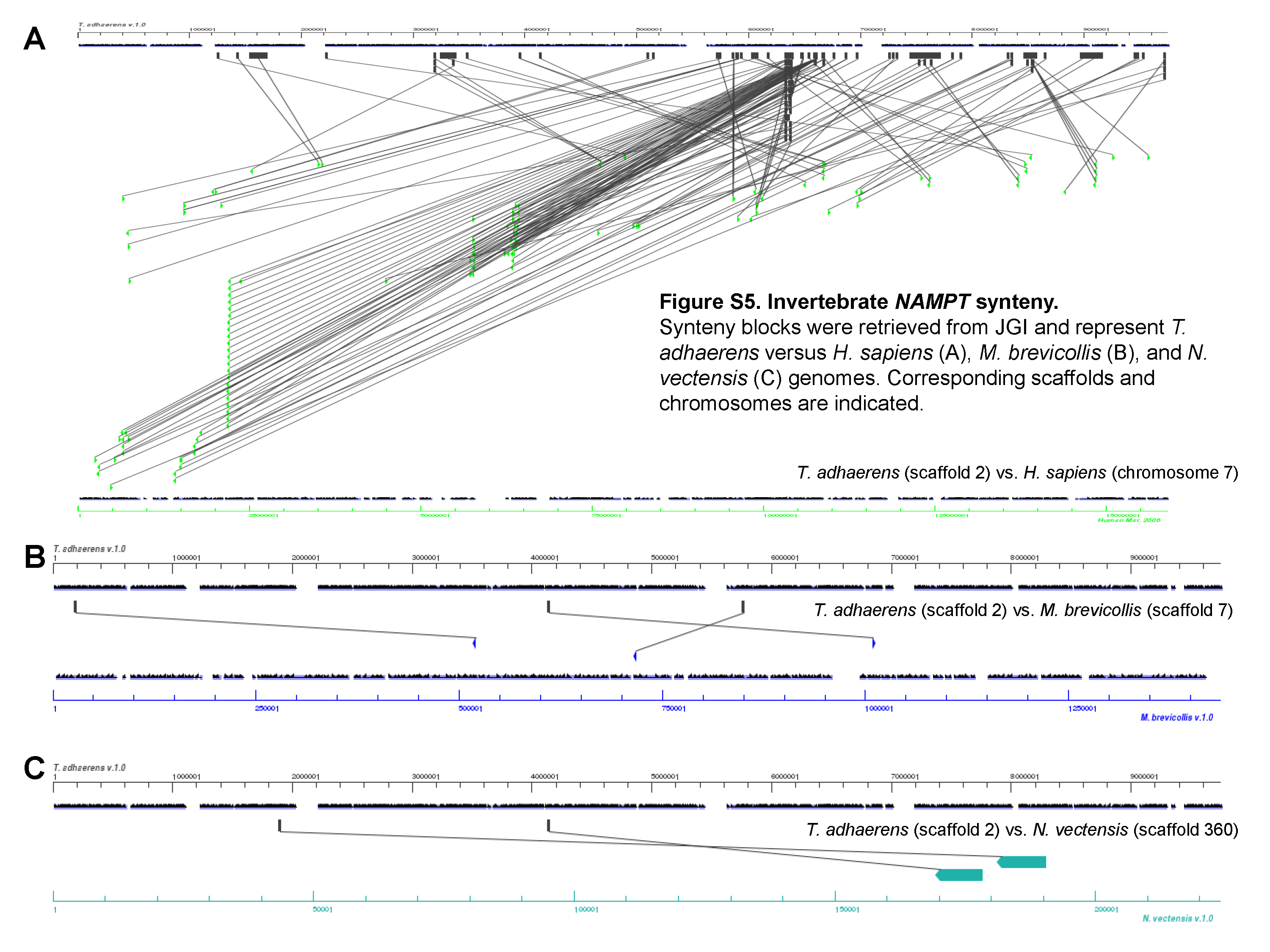

Supplement: Figure S5 — Invertebrate NAMPT synteny. Synteny blocks were retrieved from JGI and represent T. adhaerens versus H. sapiens (A), M. brevicollis (B), and N. vectensis (C) genomes. Corresponding scaffolds and chromosomes are indicated. (TIF) [file pone.0064674.s006.tif]

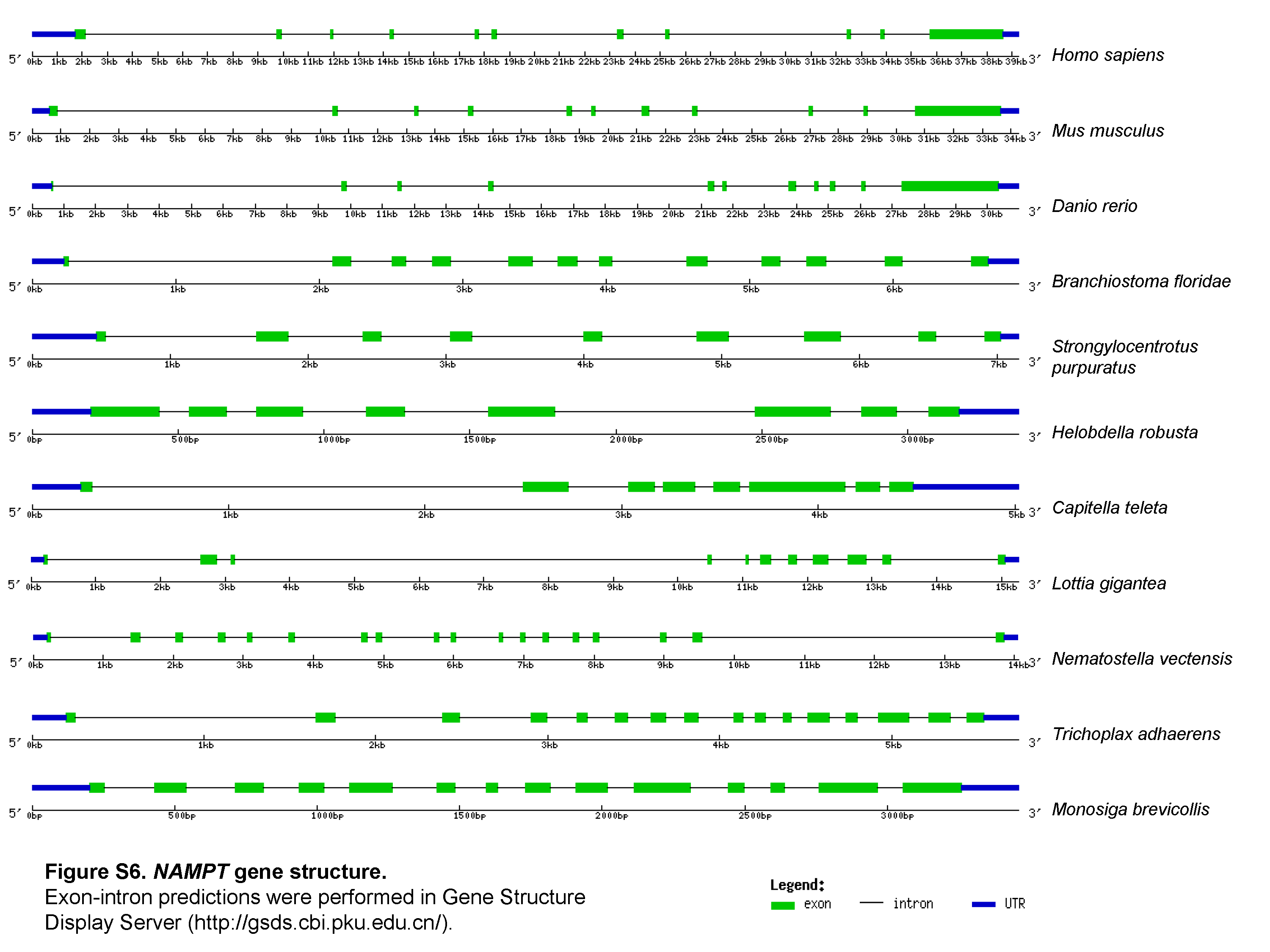

Supplement: Figure S6 — NAMPT gene structure. Exon-intron predictions were performed in Gene Structure Display Server (http://gsds.cbi.pku.edu.cn/). (TIF) [file pone.0064674.s007.tif]

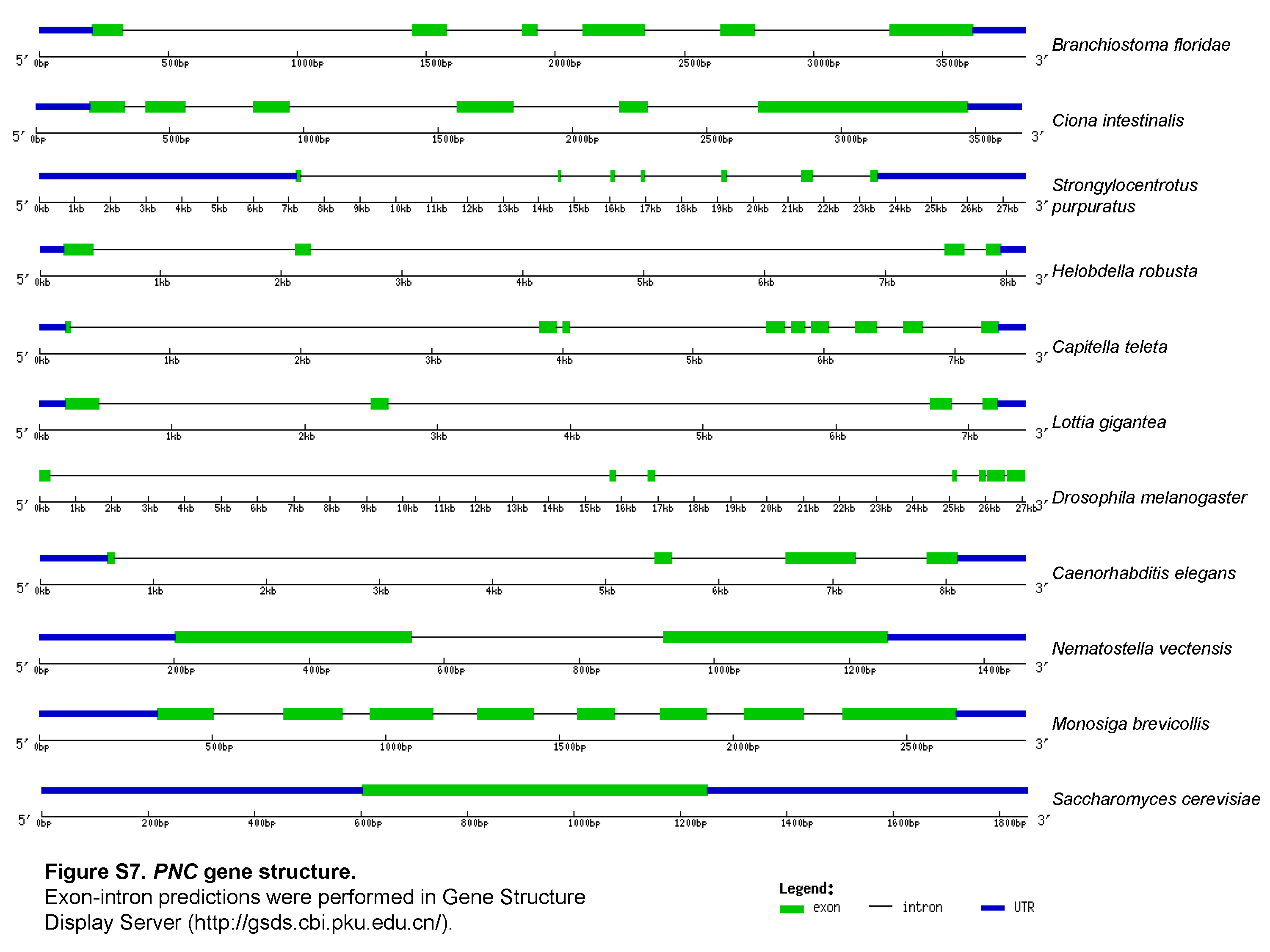

Supplement: Figure S7 — PNC gene structure. Exon-intron predictions were performed in Gene Structure Display Server (http://gsds.cbi.pku.edu.cn/). (TIF) [file pone.0064674.s008.tif]

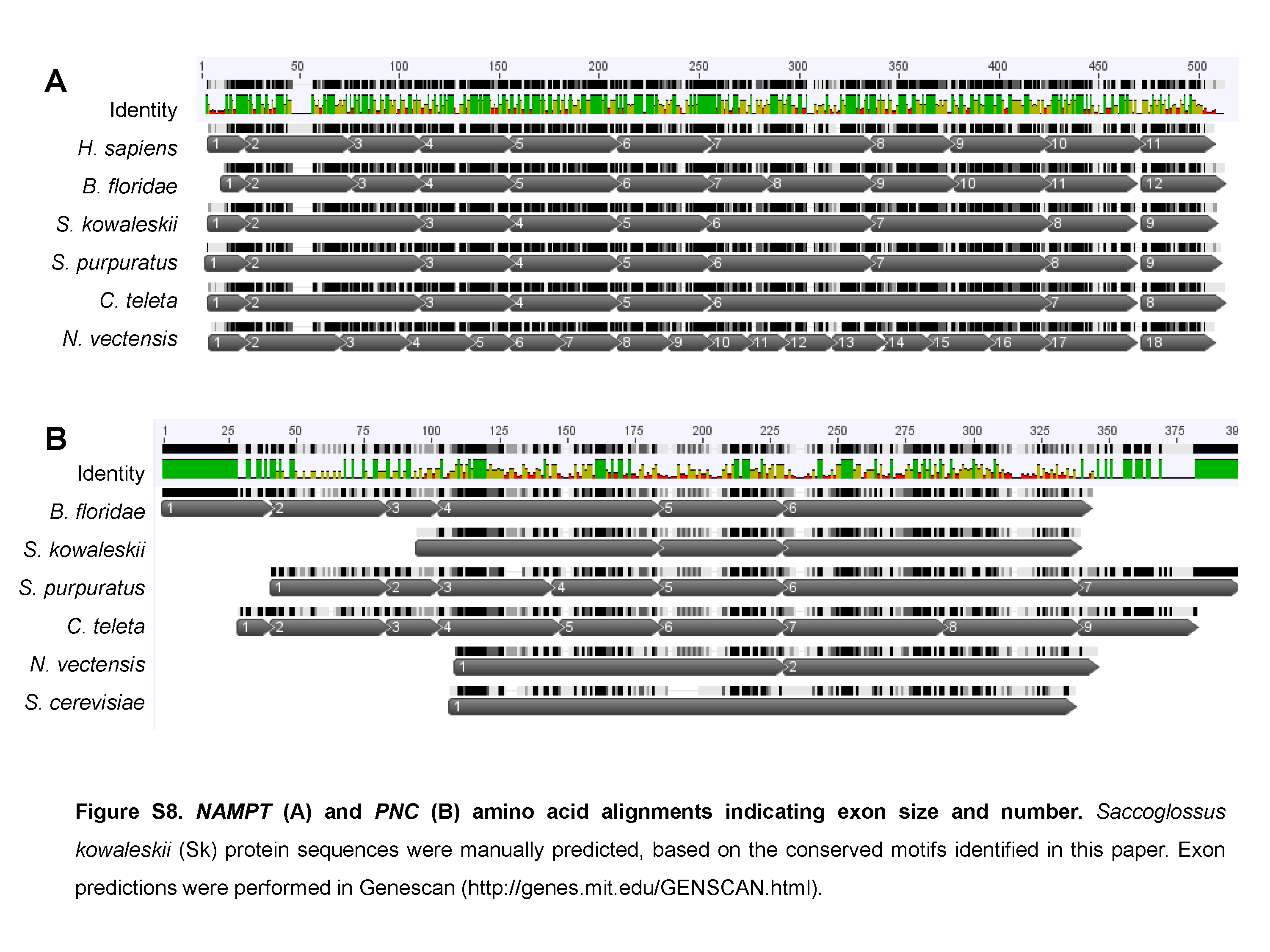

Supplement: Figure S8 — NAMPT (A) and PNC (B) amino acid alignments indicating exon size and number. Saccoglossus kowaleskii (Sk) protein sequences were manually predicted, based on the conserved motifs identified in this paper. Exon predictions were performed in Genescan (http://genes.mit.edu/GENSCAN.html). (TIF) [file pone.0064674.s009.tif]

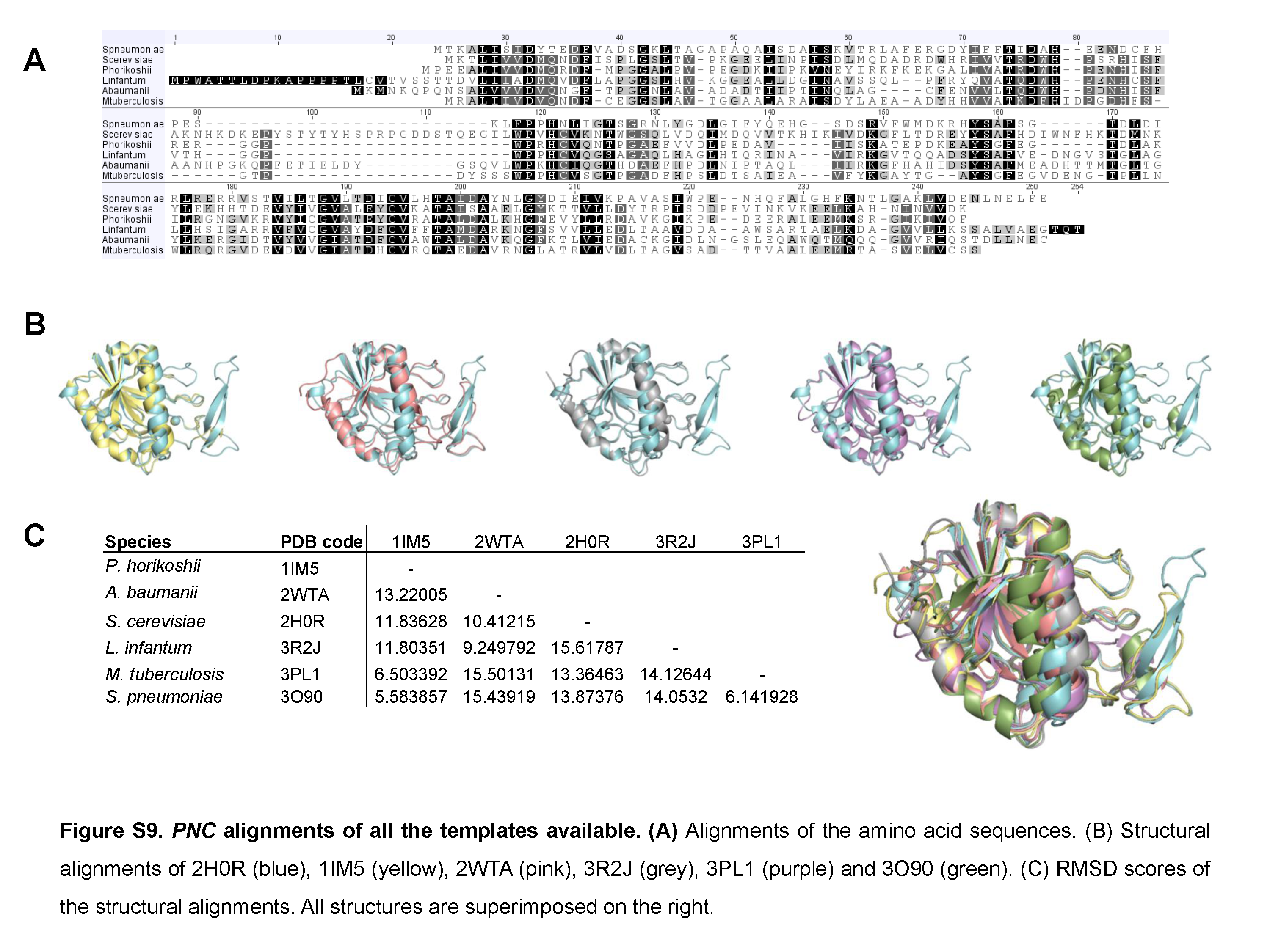

Supplement: Figure S9 — PNC alignments of all the templates available. (A) Alignments of the amino acid sequences. (B) Structural alignments of 2H0R (blue), 1IM5 (yellow), 2WTA (pink), 3R2J (grey), 3PL1 (purple) and 3O90 (green). (C) RMSD scores of the structural alignments. All structures are superimposed on the right. (TIF) [file pone.0064674.s010.tif]
